# Challenges in evaluating Welfare to Work policy interventions: would an RCT design have been the answer to all our problems?

**DOI:** 10.1186/1471-2458-10-254

**Published:** 2010-05-17

**Authors:** Kathryn Skivington, Gerry McCartney, Hilary Thomson, Lyndal Bond

**Affiliations:** 1 MRC Social and Public Health Sciences Unit, 4 Lilybank Gardens, Glasgow, G12 8RZ, UK

## Abstract

**Background:**

UK policy direction for recipients of unemployment and sickness benefits is to support these people into employment by increasing 'into work' interventions. Although the main aim of associated interventions is to increase levels of employment, improved health is stated as a benefit, and a driver of these interventions. This is therefore a potentially important policy intervention with respect to health and health inequalities, and needs to be validated through rigorous impact evaluation.

We attempted to evaluate the Pathways Advisory Service intervention which aims to provide employment support for Incapacity Benefit recipients, but encountered a number of challenges and barriers to evaluation. This paper explores the issues that arose in designing a suitable evaluation of the Pathways Advisory Service.

**Discussion:**

The main issues that arose were that characteristics of the intervention lead to difficulties in defining a suitable comparison group; and governance restrictions such as uncertainty regarding ethical consent processes and data sharing between agencies for research. Some of these challenges threatened fundamentally to limit the validity of any experimental or quasi-experimental evaluation we could design - restricting recruitment, data collection and identification of an appropriate comparison group. Although a cluster randomised controlled trial design was ethically justified to evaluate the Pathways Advisory Service, this was not possible because the intervention was already being widely implemented. However, this would not have solved other barriers to evaluation. There is no obvious method to perform a controlled evaluation for interventions where only a small proportion of those eligible are exposed. Improved communication between policymakers and researchers, clarification of data sharing protocols and improved guidelines for ethics committees are tangible ways which may reduce the current obstacles to this and other similar evaluations of policy interventions which tackle key determinants of health.

**Summary:**

The evaluation of social interventions is hampered by more than their suitability to randomisation. Data sharing, participant identification and recruitment problems are common to randomised and non-randomised evaluation designs. These issues require further attention if we are to learn from current social policy.

## Background

In 1999 the Acheson report highlighted the lack of evidence around interventions to reduce health inequalities, and recommended that all policies which impact on determinants of health, such as income, housing and education, be assessed for their health impact [1]. Ten years later an inquiry by the House of Commons Health Committee (UK) into health inequalities found that Acheson's recommendations had not generated the hoped-for evidence. The Committee concluded that the lack of evidence was "*in large part due to inadequate evaluation of the policies adopted*" [2]. Interventions which affect employment and income, such as tax and welfare policies, are an obvious possible means of improving the health of the worst off and reducing health inequalities [3-6]. Yet this appears to be a particularly neglected policy area with respect to knowledge of health impacts [7].

We set out to design an evaluation of the health impact of the Pathways Advisory Service (PAS). In our attempt to evaluate this aspect of the UK Government's Welfare to Work programme for Incapacity Benefit (IB) recipients, we struggled with the design because of the way in which the policy was implemented, because of certain characteristics of the intervention, and also because of additional pragmatic and governance restrictions. This paper discusses these difficulties with a view to proposing solutions that may facilitate such evaluations in the future. Evaluation of these interventions is important given the potentially large, but as yet unknown, health impact for this population with poor health and relatively low income [8]. The intervention discussed here is about improving access to a service i.e. key elements of the intervention are available elsewhere, but with low uptake among target groups. Our experience is therefore relevant for interventions that seek to address health inequalities by improving access routes to services already available, and to non health-focused interventions, that may have health impacts.

### Welfare to Work policy

The Welfare to Work programme in the UK seeks to reduce the number of people claiming ill-health benefits by moving them into employment. Policies aiming to move people from worklessness into employment may be regarded as important 'healthy public policy' interventions as they have the potential to impact on health and health inequalities. Although there is a positive relationship between employment and health [9,10], the potential for health benefits for welfare recipients moving into employment is likely to be dependent on the type of work obtained, the suitability to the job, job satisfaction, and individual-level factors such as age and baseline health status [6,10,11]. There is inconclusive evidence on the work-health hypothesis for this population. Very few studies show that a move from IB to employment has a positive impact upon health. Waddell and Burton's recent review of studies looking at the relationship between work and health included a section of studies on work for sick and disabled people [10]. The reported evidence comprised expert opinions and policy documents; no data from evaluations assessing the health impacts of the interventions were reported. Reviewing social security studies they reported that there may be a positive impact on health for people who voluntarily move off benefits (all types) and into work but this was not necessarily the case for all groups and depended on the type of work [10]. It is likely that those moving from sickness benefit into work may be employed in 'below average' standards of work in terms of pay and conditions, satisfaction with work etc. when they are competing with people in full health for the same jobs [12]. In this case there is the risk that a move into work may have a negative impact on health, given the type of work obtained [10,13]. We sought to improve evidence in this important policy area for public health through the rigorous evaluation of one of the UK's Welfare to Work policy initiatives which focussed on facilitating IB recipients' return to work.

### The intervention: Pathways Advisory Service

PAS was introduced in 2006; it is part of Pathways to Work, the umbrella term for the group of welfare to work initiatives aimed at people who are claiming out of work ill health benefits. Although the main aim of associated interventions is to increase levels of employment, improved health is stated as a benefit, and a driver of these interventions. PAS places employment and benefit advisors into primary care centres (GP practices) as an attempt to engage with IB recipients, utilising the endorsement of the patient's own General Practitioner (GP) and the context of a primary care setting to facilitate discussions about returning to work. PAS is aimed at improving access to services that are available to welfare recipients elsewhere e.g. Jobcentre Plus, an agency of the Government's Department for Work and Pensions. While the service is available to all IB recipients in participating GP practices, GPs use their discretion to refer patients to PAS; referral criteria are broad and it is left to the GPs' discretion to decide who is referred. Uptake is voluntary and patients can also self-refer. About 20% of IB recipients in participating practices engage with the PAS intervention (based on the average number of IB recipients per GP practice, number of people referred to PAS, and percentage of those who are referred who are on IB). It is likely that those who are referred have relatively good health and are closer to returning to the labour market than non-referred IB recipients. An evaluation of the PAS pilot programme, commissioned by the Department for Work and Pensions (DWP), reported on processes e.g. how people found out about PAS, how GPs engaged with their patients, and what PAS meetings entailed; and descriptive information on employment and benefit outcomes. There was some discussion in the report of how a move into work had impacted upon participants' health; there were both positive and negative examples of how work affected health for this group, and these were presented qualitatively [14]. The PAS programme was rolled out before the results of the evaluation by Sainsbury *et al *(2008) had been published, and before an evaluation of health impacts could be planned. We set out to design an evaluation of the health impact of PAS using self-reported health measures, primary care records and routine hospital data collection.

### Developing an impact evaluation of Pathways Advisory Service

In our attempt to evaluate PAS, a number of the characteristics of the intervention that have been described above, made it difficult for us to design a feasible evaluation. Table 1 presents a summary of these issues and the likely impact on possible findings from an evaluation.

**Table 1:** Important sources of bias independent of study design

Intervention characteristic	Potential bias & other influences on effect estimates
Only a small proportion of the eligible IB population access the intervention	Selection - it is very difficult to get a comparison group with the same characteristics as the intervention group

Intervention is to promote uptake of a service already available elsewhere (Jobcentre Plus)	Dilution - variation in exposure to the intervention across the study sample - risk of contamination among comparison group

Intervention is targeted at socio-economically deprived population	Selection & Attrition - less likely to participate in the study - little incentive for comparison group to take part in research, withdrawals likely

GP practices volunteer to participate in the intervention	Selection - primary care service that is already motivated to promote service use among vulnerable group Generalisability - GP practices who volunteer to take part may differ from those who do not

Referral is opportunistic, referral criteria not well defined	Selection - referral decisions may vary within and between GPs

Number of potential sample within an intervention practice is unknown	Selection - unknown number of eligible IB recipients will not be referred by GP

Identification and referral of eligible IB recipients initiates the intervention, before recruitment to the evaluation study	Recall - Pre-intervention data reliant on medical notes and retrospective recall from IB recipient

Expected short term health effects likely to be small	Study powered to detect small differences in health requires unfeasibly large population. Underpowered study may produce false result.

### Study Designs Considered

A variety of study designs were considered for this evaluation (see Table 2). We first looked into the possibility of conducting a randomised controlled trial (RCT), the obvious advantages being that this method would give the most comparable intervention and comparison groups, maximising internal validity. However, we believed the individual RCT design would not have been feasible as there was an infrastructure within practices to support the intervention; there were several elements of the intervention which required the entire GP practice to be involved (e.g. the presence of the PAS advisor and the use of advertising posters in the waiting room). It would also require major commitment by GPs and GPs might not recruit into the study those they thought were most in need of the service. Additionally, it could make the intervention unsustainable by reducing the referral rates to PAS, as comparison patients would obviously not be referred to the service. Because we were attempting to design an evaluation after roll out of the service had commenced, a cluster randomised controlled trial was also not possible. However even if it had been possible, it would not have overcome the difficulty in defining a comparison group from the IB recipients at non-participating GP practices, because only 20% of those eligible received the intervention (Table 2).

**Table 2:** Randomised controlled study options and key difficulties

Study design	Disadvantages specific to study design	Key difficulty	Outcome
Recruitment into study by GP followed by randomisation	Self-referral to PAS increases risk of contamination of comparison group	Service is available external to the study	Dilution bias Underestimate of effect
		
	GPs may refer those who they think most in need/most likely to benefit - rather than recruit to the study	Group being evaluated not representative of those using the service	
		
	This would half the flow of patients being referred to PAS	PAS may not be sustainable	
		
	Requires high levels of co-operation from GP and PAS	Resource implications for GPs/PAS	

Cluster randomisation	Need to identify IB recipients in comparison practices who would be eligible for referral to PAS: it is likely that this would only be around 20% of the total sample	Non-specific criteria for referral to service limits our capacity to identify an appropriate comparison group	Possible selection bias depending on ability to match controls
	
	Cluster level differences need to be accounted for	Requires high levels of collaboration with policy makers well before implementation of pilot	Not possible given that PAS had been rolled out by the time of this evaluation

The use of well conducted non-randomised study designs has been advocated as an appropriate alternative and can generate best available evidence where RCTs are not feasible [15-17]. We attempted to design a robust, quasi-experimental study: a non-randomised controlled trial. To control for important confounding factors, we considered it essential to have a comparison group in the evaluation. A suitable comparison group would have to be similar with respect to eligibility for the intervention i.e. be in receipt of IB, and also similar in terms of health status, length of time on benefits, employment history, as well as income, age, and engagement with other employment/welfare services, such as other aspects of Pathways to Work.

It is clear from Table 3 that evaluation of PAS is limited by more than simply an inability to randomise. Each of the options presented an attempt to include a comparison group - which is preferable to a simple before and after study. None of these three options provide a method for an entirely unbiased comparison group, but option 3 has the most potential. With option 2, there would have been significant issues relating to determining the comparison group by asking GPs in control practices to tell us who they think would be eligible. These issues include: the broad referral criteria would have made it difficult to ask non-PAS practices to easily identify a possible comparison group (referral criteria is likely to vary between GP practices, especially between those which have an established service and GP practices which do not offer the service at all); the fact that referral to PAS by GPs is often opportunistic; and that it would require high levels of input from GPs in non PAS practices. Instead, option 3 utilises two comparison groups: one from non-intervention practices and the other from IB recipients who are exposed to, but do not receive the intervention. This would allow us to estimate possible differential characteristics of those who engage with the service and those who were exposed but who do not engage, as well as an estimate of the effect of PAS.

**Table 3:** Non-randomised controlled study options and key difficulties

Study design	Disadvantages specific to study design	Key difficulty	Outcome
Option 1: Intervention group: engage^a ^with PAS Comparison group: active exposure but no engagement with PAS^b^	Systematic difference between intervention and comparison group. IB recipients who engage with PAS are likely to be healthier and closer to a return to work than those who are aware of PAS but are not referred (by GP or self).		Selection bias leading to overestimate of health effects

Option 2: Intervention group: engage with PAS^a^ Comparison group: not exposed to PAS^ d^	Need to identify suitable comparison group with respect to eligibility for referral to PAS (only 20% of those in the comparison GP practices would be 'comparable' to the intervention group)	How to determine suitable controls: option to ask GP in comparison practices to tell us who, in principle, they would refer to PAS. This requires a high level of involvement by GPs not offering PAS	Possible selection bias depending on ability to match controls
			
	Cluster level influences need to be accounted for		

Option 3: Intervention group: engage with PAS^a^ Comparison groups: (1) Not exposed to PAS^d^ (2) passive exposure to PAS^c^	Need to identify suitable comparison group with respect to eligibility for referral to PAS (only 20% of those in the comparison GP practices would be 'comparable' to the intervention group)	How to determine suitable controls (this will be aided by information from the group who are exposed but do not engage)	Possible selection bias depending on ability to match controls
			
	Cluster level influences need to be accounted for		

^a ^Engagement with PAS: actually met with a PAS advisor

^b ^Active exposure to PAS: GP refers patient to PAS but patient does not take up the referral

^c ^Passive exposure to PAS: Registered patient with primary care practice participating in PAS service - may or may not be aware of PAS

^d ^Not exposed to PAS: Registered patient with primary care practice not participating in PAS service

### Pragmatic and governance restrictions to the study

We initially discussed with the DWP to identify IB recipients from their records. The provision of these data was not possible due to the implementation of a data transfer ban following the high profile loss of sensitive personal information by a number of Government departments. Regardless of having these data we would still have faced issues in evaluating PAS as it would have been necessary to recruit GP practices to the study to determine who of the IB recipients were exposed to PAS, and who engaged. We therefore proposed to identify the target population of IB recipients through GP electronic records which are coded indicating a GP has completed a medical reference for an IB claim. Although unlikely to be comprehensive in their coverage of IB patients (we estimated from one practice that around 70% of those claiming IB are recorded on GP records), this approach would have provided a method of identifying potential study participants.

**Figure 1:**
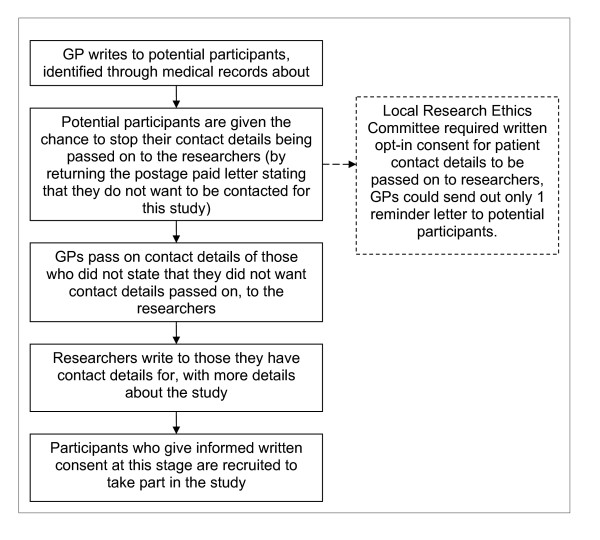
**Flowchart of proposed method of contact and consent of potential participants**.

We proposed to use an 'opt-out' method to obtain contact details of potential participants. Those who had not 'opted-out' would be invited to participate in the study, at which point we would obtain informed written consent (Figure 1).

However, the local Research Ethics Committee (LREC) and the MRC Regulatory Support Centre, advised that opt-out permission to obtain contact details was not compliant with the Data Protection Act (Figure 1). Compliance with the Act required the primary care provider to obtain written consent from patients to pass on their contact details. This meant that written consent would have to be obtained twice. For the comparison group this would be first by postal invitation to allow the GP to pass on contact details of potential participants to us, the researchers, and a second time to participate in the study. For the intervention group the PAS employment advisor would ask for the first consent, and we would obtain second written consent to include them in the study. We believed that these processes would have resulted in very low enrolment rates to the study, decreasing the genearalisabilty of the findings. Different recruitment options for intervention and comparison group would increase the likelihood of there being important differences between the groups, as the comparison group would comprise IB recipients who pro-actively volunteered to be part of a study, thus increasing the likelihood that we make a Type 1 error. To date, the obstacles identified have prevented us from undertaking an evaluation of the health effects of PAS.

## Discussion

A robust evaluation of the health impacts of PAS was severely limited because of specific intervention characteristics that made it difficult to define an appropriate comparison group; because we had no control over its roll-out; and because of pragmatic and governance restrictions leading to constrained recruitment options. It is worth considering how our experience can contribute to knowledge about the future development of evaluation methods. We believe that if the issues are not dealt with, ability to generate evidence for important policy questions and for healthy public policy will remain limited.

### Would an RCT design have been the answer?

It is acknowledged in the field of social interventions that use of randomisation in evaluation design may not always be possible, feasible [15,18,19], or appropriate given the existing evidence of known effects [16,17]. In evaluating PAS a cluster randomised trial was not feasible at this stage, although it would have been justified with respect to equipoise and the need for evidence (i.e. there is uncertainty about the harms and benefits of the intervention with respect to the intervention itself, employment, welfare, income and health). The DWP are not averse to considering and undertaking RCTs, and are currently participating in a large-scale RCT to evaluate the effectiveness of an intervention for lone parents and the long-term unemployed (although unfortunately not measuring health effects) [20]. A similar design may have been plausible to evaluate PAS, if it had been designed at the outset. However, a randomised design, developed at the stage we became involved in the evaluation, would not have been able to resolve other evaluation issues. Participants would still have to be identified through GP practices, thereby retaining the identification and recruitment problems.

### Moving towards an evaluation of the health effects on PAS

Employment and income are key determinants of health, and even marginal changes in these factors may be important for those living on low incomes and dependent on welfare. It is frustrating that policymakers may be unable to learn much about the effect on health of this policy, and that such significant barriers are in place to research with low risk to participants [8]. The challenges that we have experienced in our evaluation design are recurrent issues for researchers working in the field of healthy public policy [17,21].

Rychetnik *et al *(2002) emphasised the need to evaluate, and to use the best design appropriate to the question, but to be clear about potential for bias [22]. In reality any evaluation design can be limited by a number of factors. Where multiple concurrent limitations are experienced as we have described, this represents such a barrier, or allows only evaluation that is likely to yield data so compromised, that there is likely to be a missed opportunity to learn from new policy. We have identified multiple (potential) sources of bias in the evaluation of PAS, which leads us to contemplate whether the evaluation would be worth doing at all. Our experience has highlighted issues concerning control over implementation of the intervention, identification and transfer of contact details of potential participants, and the identification of a comparison group. There is no straightforward solution to resolve these issues, but possible routes to deal with them may include: improving the working relationship between researchers and policymakers to ensure social interventions can be evaluated, and such evaluations contribute appropriately to policy and practice; and clarification of the Data Protection Act so that it is not left open to varying ethical and legal interpretations.

#### *Closer working between policymakers and researchers*

Closer working relationships between researchers and policymakers may provide opportunities for researchers to be involved in the early stages of the development of interventions so that implementation and evaluation are not considered as separate processes. The design of appropriate evaluations would be facilitated if discussions between policymakers and evaluators began in or before what trialists refer to as phases I and II of an evaluation [23]. Discussions at these early stages may facilitate use of RCTs, but closer relationships between researchers and policymakers remain important even when randomisation is not an option. Political culture may make policymakers want to be seen to be doing 'something' and press ahead with roll out of interventions like PAS before thinking about evaluation, however as long-stated by the British Government [24], and advocated in the recent Health Select Committee report [2], it is important that policy interventions should be evaluated. Closer working relationships thus have the potential to foster an evaluation culture and an appreciation of the sometimes divergent agendas of each group, and the complexity of 'evidence based policy' given that 'evidence' may not only be based on scientific research [25].

#### *Data sharing protocols, legal and ethical interpretation of the Data Protection Act*

There is ongoing debate about the interpretation of the Data Protection Act with respect to research [26-28]. Guidance from the Information Commissioner's Office (ICO) suggests that as a data controller, an organisation should carefully consider which organisations they pass information to and should ensure the external organisation can work in a secure way with an agreed written contract [29]. In terms of organisations requesting information about individuals from a data controller, the ICO stipulates that the individuals should be informed of disclosure, but it does not stipulate that written consent is required to pass on contact details. The ICO goes on to say that "*there are a number of exemptions that allow disclosure in certain circumstances*"; however it is unclear what they are.

The Data Protection Act is open to interpretation, and its interpretation would appear to vary between Research Ethics Committees. There is a recent example of a research study successfully gaining ethical approval to use the 'opt out' contact approach that we originally proposed for this study [30]. GP practices were recruited, potential participants were identified and written to by their GP and informed that their contact details would be passed on to the researchers unless they replied stating that they did not wish to take part. Researchers then contacted the potential participants by telephone to recruit them to the study.

In our study, the requirement to obtain written consent for contact details to be passed to researchers, and again at the point of study recruitment, was likely to lead to decreased external validity. The recruitment rates reported for other studies with 'opt in' consent before the researchers can approach the potential participants is between 8% and 34% [31-34]. Furthermore, response rates among deprived populations, such as IB recipients, are known to be low [35,36]. Given these facts, we were sceptical about the ability to recruit a suitably representative sample, which led us to question the study's potential value.

There is an obvious tension between the competing interests of individual privacy and research [27,28,37,38]. We support the importance placed on individual privacy, particularly for vulnerable groups such as IB recipients who are already subject to considerable levels of surveillance [39]. However, it can also be argued that continued implementation of policies that are not properly evaluated is unethical and permits potentially damaging interventions for vulnerable groups. Under present arrangements, evaluation of interventions such as PAS will be undermined by a recurring recruitment bias caused by lack of an accessible sample frame, and evaluations will be limited to internal evaluations or ecological-level analyses of routine population data. There does not appear to be standardised guidance for research ethics committees on how to deal with requests such as ours and it may be that more transparent guidance would assist both researchers and ethics committees. If possible, the guidance would take into account the need for research and evaluation, whilst protecting participants' rights. This would allow researchers to know what is considered ethical and also what procedures are required to comply with legislation while designing a study. It is important to bear in mind that ethical approval does not mean that the research is legally in compliance with the Data Protection Act; therefore development of standard ethical guidelines which take this Act into account would rely on clarification of the Act itself.

## Summary

Health is a key consideration in Welfare to Work policies, and it is widely cited as part of the rationale for returning IB recipients to work, yet the relative benefits and harms of PAS are unknown. There is a tension between the ethical requirements to protect the privacy of the individual and the need to assess, through evaluation, the actual impacts of the intervention. If the potential health effects of these policies are to be realised it is essential that programmes designed to implement them can be rigorously evaluated to determine both positive and negative effects. Even where a RCT is appropriate, limitations to evaluation may remain. Our experience is that evaluations, particularly after the intervention has been implemented, remain limited by: a lack of suitable methods for evaluating interventions where only a small proportion of the eligible population will engage; data sharing prohibitions; privacy rules; and the interpretation of such rules by ethics committees. These problems are not new but are likely to continue to impede the development of evidence-based policy until action is taken.

## Competing interests

The authors declare that they have no competing interests.

## Authors' contributions

All authors worked on the planning for the primary study discussed in this manuscript. KS drafted the manuscript. GM, HT and LB contributed to the writing of the paper and provided edits. All authors read and approved the final manuscript.

## Funding

Kathryn Skivington, Gerry McCartney, Hilary Thomson and Lyndal Bond are funded by the Chief Scientist Office at the Scottish Government Health Directorate as part of the Evaluating Social Interventions programme at the MRC Social and Public Health Science Unit, (U.130059812)

## Pre-publication history

The pre-publication history for this paper can be accessed here:

http://www.biomedcentral.com/1471-2458/10/254/prepub
